# Gene Mining for Conserved, Non-Annotated Proteins of *Podosphaera xanthii* Identifies Novel Target Candidates for Controlling Powdery Mildews by Spray-Induced Gene Silencing

**DOI:** 10.3390/jof7090735

**Published:** 2021-09-08

**Authors:** Laura Ruiz-Jiménez, Álvaro Polonio, Alejandra Vielba-Fernández, Alejandro Pérez-García, Dolores Fernández-Ortuño

**Affiliations:** 1 Departamento de Microbiología, Facultad de Ciencias, Universidad de Málaga, 29071 Málaga, Spain; laura110493@uma.es (L.R.-J.); polonio@uma.es (Á.P.); avielba@uma.es (A.V.-F.); aperez@uma.es (A.P.-G.); 2 Instituto de Hortofruticultura Subtropical y Mediterránea “La Mayora”-Universidad de Málaga-Consejo Superior de Investigaciones Científicas (IHSM-UMA-CSIC), 29071 Málaga, Spain

**Keywords:** cucurbits, dsRNA, *Podosphaera xanthii*, powdery mildews, RNAi, spray-induced gene silencing (SIGS)

## Abstract

The powdery mildew fungus *Podosphaera xanthii* is one of the most important limiting factors for cucurbit production worldwide. Despite the significant efforts made by breeding and chemical companies, effective control of this pathogen remains elusive to growers. In this work, we examined the suitability of RNAi technology called spray-induced gene silencing (SIGS) for controlling cucurbit powdery mildew. Using leaf disc and cotyledon infiltration assays, we tested the efficacy of dsRNA applications to induce gene silencing in *P. xanthii*. Furthermore, to identify new target candidate genes, we analyzed sixty conserved and non-annotated proteins (CNAPs) deduced from the *P. xanthii* transcriptome in silico. Six proteins presumably involved in essential functions, specifically respiration (CNAP8878, CNAP9066, CNAP10905 and CNAP30520), glycosylation (CNAP1048) and efflux transport (CNAP948), were identified. Functional analysis of these CNAP coding genes by dsRNA-induced gene silencing resulted in strong silencing phenotypes with large reductions in fungal growth and disease symptoms. Due to their important contributions to fungal development, the *CNAP1048*, *CNAP10905* and *CNAP30520* genes were selected as targets to conduct SIGS assays under plant growth chamber conditions. The spray application of these dsRNAs induced high levels of disease control, supporting that SIGS could be a sustainable approach to combat powdery mildew diseases.

## 1. Introduction

Powdery mildew diseases are undoubtedly some of the most important plant diseases worldwide [1]. Major crops, including cereals, grapevines and a number of economically important vegetables and ornamentals, are among their targets [2,3,4]. These diseases are caused by powdery mildew fungi (*Ascomycota*, *Erysiphales*), which are obligate biotrophic plant pathogens that can only grow and reproduce on living host tissues to complete their life cycles. In general, these fungi show both asexual and sexual phases. The asexual part starts after a conidium lands on a susceptible host plant. Then, the conidium germinates and produces a germ tube, ending in a primary differentiated appressorium, from which a primary haustorium is developed. This specialized structure of parasitism is responsible to uptake nutrients and exchange other factors with the plant cell. If the infection is successful, a primary hypha arises that forms secondary hyphae, from which conidiophores emerge vertically, producing a variable number of conidia. Under favorable environmental conditions (low humidity and temperatures between 21–25 °C), this epiphytic fungal growth results in typical disease symptoms, a visible white powdery mass on plant surfaces. When the sexual phase occurs, two hyphae of opposite mating types must merge and form a chasmothecium; however, in the case of some species, chasmothecia are rarely observed in the field [5,6,7]. Owing to their nature as obligate parasites, they cannot be cultured on nutrient media, a fact that has significantly hampered research [5]. Various horticulture crops are susceptible to powdery mildews, but cucurbits are arguably the group more severely affected. *Podosphaera xanthii* is the principal causal agent of cucurbit powdery mildew disease and one of the most important limiting factor for crop productivity of fungal origin [8,9,10]. Chemical control and the use of resistant cultivars are the main tools for cucurbit powdery mildew management. Unfortunately, the rapid development of new physiological races [9] and the emergence of isolates resistant to the most commonly used anti-powdery mildew fungicides [11] have resulted in a situation where, disease control remains elusive to growers. For this reason, novel disease management strategies are, more than ever, needed.

RNAi is well-known as a preserved and integral part of the gene regulation process present in all eukaryotes and is regulated by small RNAs (sRNAs) that direct gene silencing at the posttranscriptional level. The cellular RNAi machinery starts with double-stranded RNAs (dsRNAs), which are initially processed into small interfering RNAs (siRNAs) by an RNaseIII-like enzyme called Dicer, leading to the degradation of target mRNAs across the action of the RNA-induced silencing complex (RISC), resulting in the knockdown of protein expression [12]. Recent works have found that fungal pathogens such as *Botrytis cinerea*, *Fusarium graminearum* and *Sclerotinia sclerotium* can efficiently catch environmental dsRNAs, which are modified into siRNAs and lead the silencing of pathogen genes with complementary sequences [13,14,15]. These discoveries encouraged the development of a novel crop protection strategy designated spray-induced gene silencing (SIGS) that involves the exogenous application of dsRNAs onto plants to silence essential pathogen genes [16,17]. This was the case for the Asian soybean rust fungus *Phakopsora pachyrhizi*; a substantial reduction in infection was shown after spraying dsRNAs targeting three genes involved in urediniospore germination or appressorium formation [18]. The effectiveness of SIGS has been studied across a wide range of fungal pathogens [19]; however, there are no reports describing the potential of SIGS against powdery mildew fungi.

In recent years, remarkable efforts have been invested in deciphering the molecular bases of *P*. *xanthii* biology. The current development of genomic, transcriptomic, and proteomic technologies has conceded the production of fundamental resources for *P. xanthii* research, such as the epiphytic and haustorial transcriptomes [20,21,22] and the first draft genomes [23,24], as well as RNA-seq analyses of the early stages of infection in melon and other hosts [25,26,27]. In addition, specific tools for the functional analysis of *P. xanthii* genes, such as transformation and RNAi silencing protocols, have been developed [5,28,29,30], which have allowed the discovery of the first *P. xanthii* effectors [30,31,32]. Despite this substantial progress, further research on *P. xanthii* is still needed to uncover novel genes and pathways that allow us to understand the singularities of this important pathogen of cucurbits and, eventually, to develop new control strategies.

The aims of this work were (i) to explore the potential of RNAi technology for cucurbit powdery mildew management by SIGS and (ii) to identify new target candidate genes for this technology. For these purposes, we first developed a new and simple gene silencing method for *P. xanthii* based on the application of dsRNAs to the plant surface. To search for new targets, we focused on the study of a set of 60 conserved and non-annotated proteins (CNAPs) of *P. xanthii*. After protein modeling and ligand prediction studies, we selected six *P. xanthii* genes with putative functions in respiration, glycosylation and efflux transport to be analyzed by dsRNA-induced gene silencing. Our results showed that these genes are essential for *P. xanthii* development and potential targets for RNAi-based approaches and that SIGS could be a promising tool for controlling powdery mildews.

## 2. Materials and Methods

### 2.1. Plants, Microbes and Culture Conditions

Cotyledons of zucchini (*Cucurbita pepo* L.) cv. Negro Belleza (Semillas Fitó, Barcelona, Spain) and plants of melon (*Cucumis melo* L.) cv. Rochet (Semillas Fitó) were used for fungal growth and dsRNA assays, respectively. Plants were grown in growth chambers at 24 °C under a 16 h light/8 h dark photoperiod. The *P. xanthii* isolate 2086 was used in this study. For the growth of the fungal isolate, disinfected cotyledons of zucchini maintained in vitro in 8-cm Petri dishes with Bertrand medium were used as previously described [33]. For the maintenance, construction, and propagation of RNAi silencing vectors, *Escherichia coli* strain DH5α was used. The strain was routinely grown at 37 °C in lysogeny broth (LB) medium with ampicillin (100 µg mL^−1^) when required. 

### 2.2. Sequence Analysis, Protein Modeling and Protein Function Prediction

The identification of conserved and non-annotated proteins (CNAPs) was conducted using the comprehensive transcriptome of *P. xanthii*. This transcriptome analysis was performed with TransFlow [34] by combining the *P. xanthii* epiphytic [20] and haustorial [22] raw reads and annotating them against Kingdom Fungi orthologues from UniProtKB using FullLengtherNEXT software (http://www.scbi.uma.es/fulllengthernext accessed on 12 July 2021). To identify the CNAPs, complete proteins without functional annotation or known domains and with an identity higher than 75% with other fungi were selected from the FullLengtherNEXT annotation file. To confirm the lack of function and the high identity with other fungi of these proteins, BLASTp (BLAST+ v.2.7.1) analysis against the NCBI-nr database (E-value > 1 × 10^−5^) was carried out. The MEGA X software tool was used to generate phylogenetic trees [35], whereas the UniProt server [36] was used to retrieve protein sequences from the Protein Data Bank (PDB). In addition, for CNAP identification, only nonsecreted proteins were considered. For this purpose, the web servers Secretool, PECAS secretome (to predict secreted proteins) [37,38], Deeploc (to predict the exact subcellular location of the proteins) [39] and PredGPI (to predict hypothetical GPI-anchor domains) [40] were used. Only those proteins with a concordant cytoplasmic location according to all software were selected.

Finally, to gain insight into the putative functions of CNAPs, the webserver I-TASSER [41] was employed to perform automated protein structure homology modeling by fold recognition searches using the crystal structures of proteins with known functions available in the PDB database. The measure of the quality of a predicted structure is its estimated C (confidence) and TM (template modeling) score values. According to I-TASSER, C score values in the range between −5 and 2 indicate a more confident model, and TM score values >0.5 indicate a correct topology. Additional software tools used to obtain more information about the putative functions of CNAPs were Motif Scan (to predict domains in the mature proteins) [42], 3DLigandSite, COACH and CATH/Gene3D (to predict ligands) [43,44,45].

### 2.3. Isolation of Nucleic Acids and cDNA Synthesis

To isolate DNA and RNA from *P. xanthii*-infected melon cotyledons, the cotyledons were frozen in liquid nitrogen and stored at −80 °C until use. The frozen cotyledons were ground with a mortar and pestle. Genomic DNA was isolated using the MasterPure^TM^ Yeast DNA Purification Kit (Epicentre Biotechnologies, Madison, WI, USA), and total RNA was extracted using TRI Reagent (Sigma-Aldrich, Steinheim, Germany) following the manufacturer’s instructions. Total RNA was eluted in diethyl pyrocarbonate (DEPC)-treated water and stored at −80 °C until use. The RNA concentration was estimated using a NanoDrop 2000 spectrophotometer (Thermo Fisher Scientific, Waltham, MA, USA), and cDNA synthesis was performed using Superscript III reverse transcriptase (Thermo Fisher Scientific) and random primers (Thermo Fisher Scientific) according to the manufacturer´s recommendations.

### 2.4. In Vitro Production of dsRNA

For the production of double-stranded RNA (dsRNA) molecules, an in vitro method based on cloning the target sequence of interest into a plasmid with opposite T7 RNA polymerase promoters and a one-step PCR procedure to amplify the target sequence carrying the T7 promoter sequence at both ends, followed by in vitro transcription, were used as previously described [15]. In brief, a fragment of each *P. xanthii* gene and a 379 bp fragment of the melon *CmMlo1* gene used as a control for RNAi-induced powdery mildew resistance were amplified from cDNA using Phusion^TM^ High-Fidelity DNA polymerase (Thermo Fisher Scientific) under the following conditions: one cycle of 98 °C for 30 s, 35 cycles of 98 °C for 10 s, 60 °C for 30 s and 72 °C for 30 s, and one cycle for 72 °C for 7 min. The primers used for this purpose are listed in Table S1 and were designed using Primer3 software (http://bioinfo.ut.ee/primer3/ accessed on 12 July 2021). [46]. Subsequently, PCR products were digested with FastDigest *Nco*I and *Bgl*II restriction enzymes (Thermo Fisher Scientific) and cloned into the same cut pL4440 vector (kindly donated by Andrew Fire, Stanford University) using T4 DNA ligase (Thermo Fisher Scientific) according to the manufacturer´s recommendations. The resulting plasmids were propagated and maintained in *E. coli* DH5α, and sequence inserts were verified by PCR amplification, digestion, and sequencing (Stab Vida, Caparica, Portugal). To amplify each insert flanked with T7 promoters, the pair of primers T7-F and T7-R (Table S1) was employed using the PCR conditions indicated above. Finally, each resulting PCR product was employed as a template in an in vitro transcription reaction using the MEGAScript^TM^ RNAi kit (Invitrogen, Carlsbad, CA, USA) according to the manufacturer´s instructions. The dsRNA concentration was estimated using a NanoDrop 2000 spectrophotometer (Thermo Fisher Scientific), and dsRNA integrity was confirmed by visualization on 1% agarose gels.

### 2.5. dsRNA-Mediated Gene Silencing

#### 2.5.1. Leaf Disc Assay

To test the effect of the application of exogenous dsRNAs on *P. xanthii* development, a previously reported leaf disc assay [47] was used. Briefly, melon cotyledon discs (9 mm diameter) from 8-day-old plants were incubated with 3 mL of aqueous solution containing the corresponding dsRNA and 0.03% of the surfactant Silwet L-77 (Lehle Seeds, Round Rock, TX, USA). To examine the effect of dsRNA concentration on target gene knockdown, dsRNA solutions were prepared in DEPC-treated water and adjusted to a final concentration ranging from 100 to 1000 ng mL^−1^. After 3 h of incubation, discs were aseptically transferred onto agarised medium and inoculated with 10 µL of fresh suspensions of *P. xanthii* conidia adjusted to 1 × 10^5^ conidia mL^-1^. Later, dsRNA-treated discs were maintained in a growth chamber under a 16 h light/8 h dark photoperiod at 24 °C until evaluation of disease severity by image analysis. For this, pictures were taken and processed using Fiji software [48], and disease symptoms were estimated by percentage quantification of the disc surface covered by powdery mildew. As negative controls, discs treated with dsRNA obtained from an empty vector were used.

#### 2.5.2. Cotyledon Infiltration Assay

To analyze the effects of dsRNAs on fungal development and *P. xanthii* gene expression, infiltration assays were performed. For these assays, melon cotyledons from 8-day-old plants were used as previously described [30]. Prior to each assay, dsRNA solutions were prepared in infiltration medium (10 mM MES and 10 mM MgCl_2_) and adjusted to a final concentration ranging from 100 to 1000 ng mL^−1^. Next, dsRNA solutions were infiltrated onto the abaxial leaf surface using an insulin needle-less syringe. Twenty-four hours after infiltration, melon cotyledons were inoculated with fresh suspensions of *P. xanthii* conidia (1 × 10^5^ conidia mL^−1^) by spraying and maintained in a growth chamber under the conditions shown above until microscopy and qPCR analyses were performed. As negative controls, cotyledons infiltrated with dsRNA obtained from an empty vector were used.

### 2.6. Quantitative Reverse Transcription RT-qPCR and qPCR

The expression analysis of *P. xanthii* genes and the molecular estimation of *P. xanthii* biomass were carried out by RT-qPCR and qPCR, respectively. The primers used for these analyses (Table S1) were designed using Primer3 software [46,49]. To investigate the effect of dsRNAs on the expression of *P. xanthii* and melon genes, cDNA obtained from dsRNA-infiltrated and *P. xanthii*-infected melon cotyledons at 24 h postinoculation (hpi) was used. As normalization reference genes, the *P. xanthii* translation elongation factor 1-alpha gene *PxEF1* (MK249653) and the *C. melo* actin-7 gene *CmACT7* (XM_008462689.2) were used [22,25]. For the molecular estimation of fungal biomass, total DNA isolated from dsRNA-infiltrated and infected melon cotyledons at 72 hpi were used. For this purpose, the *P. xanthii* β-tubulin gene *PxTUB2* (KC333362) and the *C. melo* actin-7 gene *CmACT7* (XM_008462689.2) were quantified, and the *P. xanthii*/*C. melo* genomic DNA ratio was calculated as previously described [50]. RT-qPCR and qPCR assays were carried out in a CFX384 Touch Real-Time PCR detection system (Bio-Rad, Hercules, CA, USA) using SsoFast EvaGreen Supermix (Bio-Rad) according to the manufacturer´s instructions with the following cycling conditions: enzyme activation step at 95 °C for 30 s, followed by 40 cycles at 95 °C for 5 s and 65 °C for 5 s. All reactions were performed in quadruplicate. After amplification, the data were processed by CFX Manager Software (Bio-Rad), and the amplicon sizes were confirmed by visualization on 2% agarose gels.

### 2.7. Haustorial Counts

The progress of *P. xanthii* infection upon infiltration of melon cotyledons with dsRNAs was evaluated by histochemical analysis according to the 3,3′-diaminobenzidine (DAB)-uptake method [51] as previously described [30]. Briefly, cotyledon discs (1 cm diameter) were incubated in 1 mg mL^−1^ DAB (pH 3.8) overnight in the dark and at room temperature. After incubation, discs were decolourized in boiling ethanol and observed by light microscopy using an Eclipse E800 microscope (Nikon, Tokyo, Japan). With these preparations, the haustoria were easily visualized as dark brown spots beneath the *P. xanthii* hyphae inside epidermal cells. Images were captured with a Leica DFC450 camera attached to the microscope and processed using the LAS-AF software LCS Lite (Leica Microsystems, Wetzlar, Germany).

### 2.8. SIGS Assay

To evaluate the efficacy of dsRNA-RNAi against *P. xanthii*, SIGS assays were carried out using 3-week-old melon plants. For this purpose, dsRNA solutions were prepared in DEPC-water and adjusted to different concentrations (5, 10, 20 and 30 µg mL^−1^). For their application on melon leaves, the corresponding dsRNAs were sprayed onto the leaf surface until the point of runoff. Next, dsRNA-treated leaves were maintained in a growth chamber under a 16 h light/8 h dark photoperiod at 24 °C for 24 h until inoculation with fresh *P. xanthii* conidial suspensions (1 × 10^4^ conidia mL^−1^) by spraying. Then, melon plants were maintained under the same conditions until evaluation of disease symptoms by image analysis as described above. As negative controls, melon leaves were sprayed with water or with dsRNA obtained from an empty vector. 

### 2.9. Statistical Analysis

When required, statistical analysis of data was carried out by IBM SPSS v. 25 software (SPSS, Chicago, IL, USA) using Fisher’s least significant difference test (LSD).

## 3. Results

### 3.1. Identification and Selection of CNAPs

From the transcriptome of *P. xanthii*, which was determined with TransFlow by combining the epiphytic [20] and haustorial [22] transcriptomes, we identified a group of sixty *P. xanthii* proteins (Table S2) without functional annotation or known domains and with an identity higher than 75% with other fungi according to FullLengtherNEXT annotation results; these proteins were designated conserved and non-annotated proteins (CNAPs). All of them were also characterized as nonsecreted proteins and by a predicted cytoplasmic location. To decipher the biological functions of these CNAPs, protein models were obtained for each protein using the I-TASSER server and the amino acid sequences of the proteins. In addition, a set of prediction tools (Motif Scan, 3DLigandSite, COACH and CATH/Gene3D) was employed for function and ligand predictions using both the amino acid sequences and the protein prediction models. From this analysis, six CNAPs were selected according to the quality of the protein models and concordance with the prediction tools. These proteins, CNAP948, CNAP1048, CNAP8878, CNAP9066, CNAP10905 and CNAP30520, showed high identities to hypothetical proteins of other powdery mildew species, such as *Oidium neolycopersici* and *Blumeria graminis*, and other filamentous fungi (Table 1).

**Figure 1:**
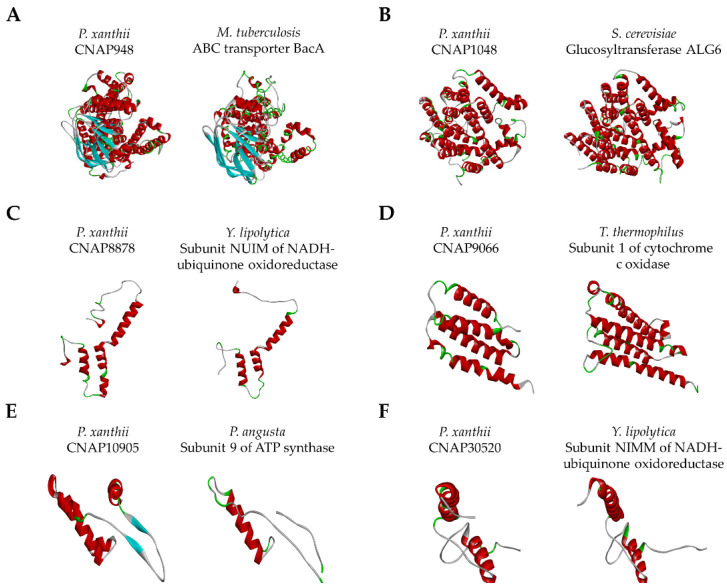
Predicted three-dimensional (3D) models of *Podosphaera xanthii* conserved and non-annotated proteins (CNAPs) and their best structural analogues. 3D models were constructed using the I-TASSER server. (**A**) CNAP948 model and the ABC transporter BacA from *Mycobacterium tuberculosis* (6TQE). (**B**) CNAP1048 model and yeast ALG6 glucosyltransferase (6SNH). (**C**) CNAP8878 model and the subunit NUIM of NADH-ubiquinone oxidoreductase from yeast (6RFQ). (**D**) CNAP9066 model and subunit 1 of cytochrome c oxidase from *Thermus thermophilus* (1XME). (**E**) CNAP10905 model and subunit 9 of ATP synthase from *Pichia angusta* (5LQX). (**F**) CNAP30520 model and the NIMM subunit of NADH-ubiquinone oxidoreductase from *Yarrowia lipolytica* (6Y79).

Regarding their putative functions, these CNAPs seem to be involved in physiological functions such as efflux transport, glycosylation or respiration (Figure 1; Table 2). The CNAP948 model presented a significant structural analogy with the ABC transporter (BacA) from *Mycobacterium tuberculosis* (PDB code 6TQE) (Figure 1A; Table 2) and ATP as a protein ligand, suggesting that CNAP948 could be a protein involved in the efflux transport of molecules. The resulting model of CNAP1048 showed structural analogy with a glucosyltransferase (ALG6) from *Saccharomyces cerevisiae* (PDB code 6SNH) and glucose as a protein ligand (Figure 1B; Table 2), suggesting that CNAP1048 might be involved in glycosylation, which is part of the posttranslational modification of proteins. The CNAP8878 model showed structural analogy with the subunit NUIM of NADH-ubiquinone oxidoreductase from *Yarrowia lipolytica* (PDB code 6RFQ). Ligand prediction indicated putative interactions with iron-sulfur clusters (Figure 1C; Table 2). According to these predictions, CNAP8878 could be involved in cellular respiration. The model of CNAP9066 showed high structural analogy with subunit 1 of cytochrome c oxidase from *Thermus thermophilus* (PDB code 1XME) and putative interactions with copper ions (Figure 1D; Table 2). These predictions suggest that CNAP9066 may also be involved in respiration. The resulting model of CNAP10905 showed significant structural analogy with subunit 9 of ATP synthase from *Pichia angusta* (PDB code 5LQX) and ADP as a protein ligand (Figure 1E; Table 2), suggesting that CNAP9066 may also be involved in respiration. Finally, the CNAP30520 model exhibited high structural analogy with the subunit NIMM of NADH-ubiquinone oxidoreductase from *Yarrowia lipolytica* (PDB code 6Y79) and putative interactions with iron-sulfur clusters (Figure 1F; Table 2), suggesting a putative function related to respiration.

**Figure 2:**
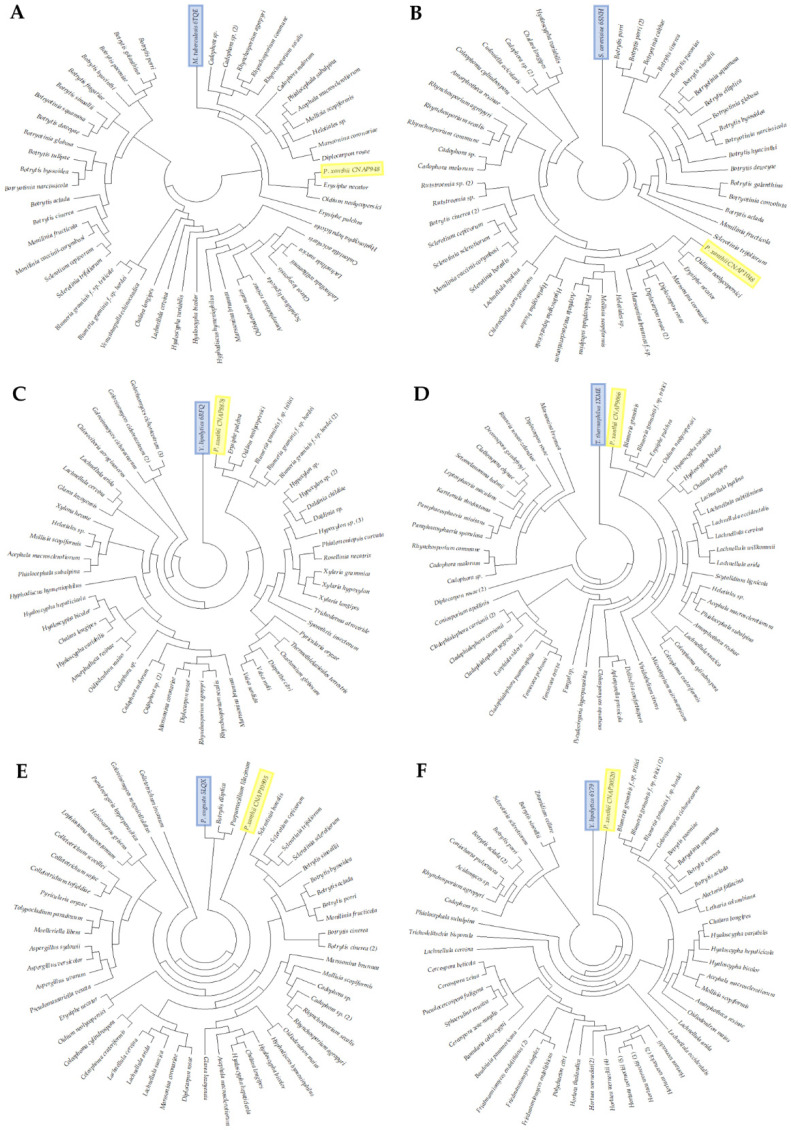
Phylogenetic analysis of six selected CNAPs. (**A**) The ABC transporter BacA from *Mycobacterium tuberculosis* (6TQE) is structurally close to 3D model of *P. xanthii* CNAP948. (**B**) Yeast ALG6 glucosyltransferase (6SNH) is structural analog to *P. xanthii* CNAP1048 model. (**C**) The subunit NUIM of NADH-ubiquinone oxidoreductase from yeast (6RFQ) is structurally close to *P. xanthii* CNAP8878 model. (**D**) Subunit 1 of cytochrome c oxidase from *Thermus thermophilus* (1XME) is structural analog to 3D model of *P. xanthii* CNAP9066. (**E**) Subunit 9 of ATP synthase from *Pichia angusta* (5LQX) is structurally close to *P. xanthii* CNAP10905 model. (**F**) The NIMM subunit of NADH-ubiquinone oxidoreductase from *Yarrowia lipolytica* (6Y79) is structural analog to 3D model of *P. xanthii* CNAP30520. *Podosphaera xanthii* CNAP proteins are labelled in yellow, and proteins structurally close to the 3D models *P. xanthii* CNAPs are shown in blue. Phylogenetic trees were constructed using the MEGA X software tool. Trees show that these CNAPs are widely distributed in fungi.

As previously mentioned, CNAPs are conserved and non-annotated fungal proteins. To demonstrate this conservation, phylogenetic trees were constructed for each of the six proteins (Figure 2). As shown in the figure, phylogenetic analysis showed that these proteins are widely present within *Ascomycota*, not only in plant pathogenic fungi but also in fungi that are insect and human pathogens, as well as in saprophytes.

### 3.2. Development of a dsRNA-Mediated Gene-Silencing Assay for P. xanthii

**Figure 3:**
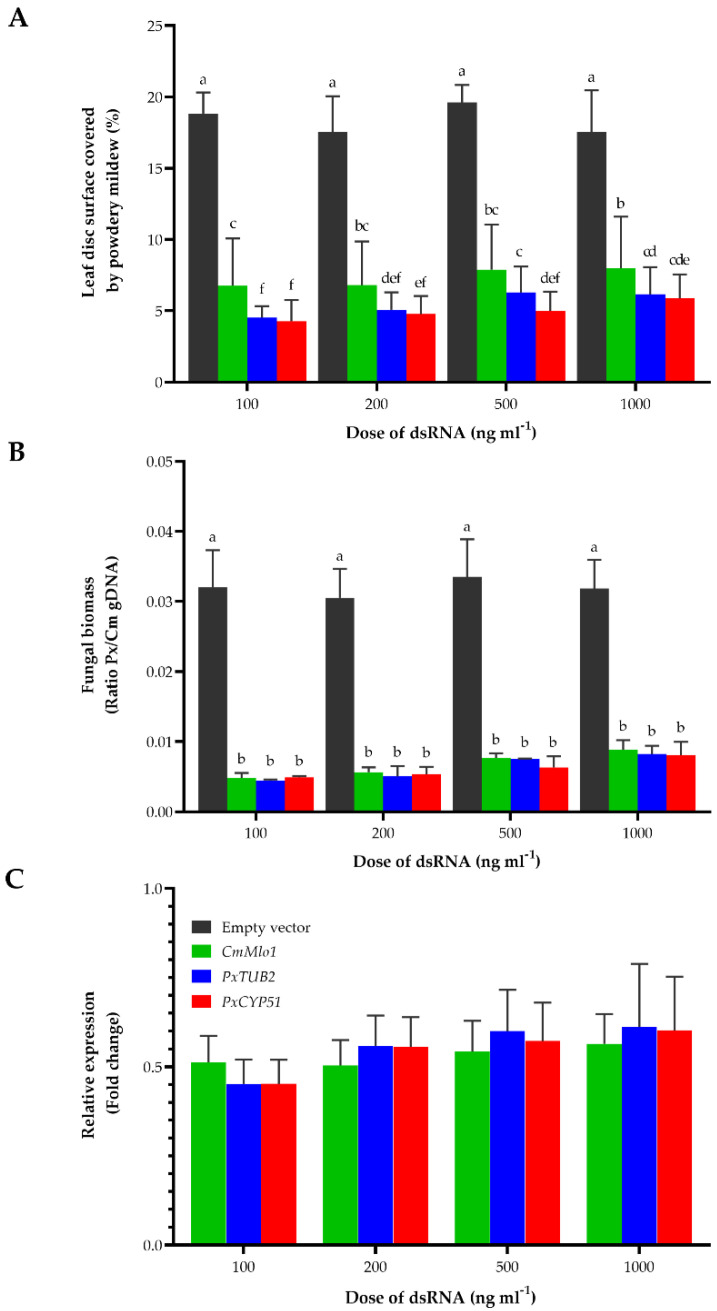
Effect of foliar application of exogenous double-stranded RNA (dsRNA) on *Podosphaera xanthii* growth and disease development. (**A**) Melon cotyledon discs were exposed to different doses of dsRNA for *CmMlo1*, *PxTUB2* and *PxCYP51* genes and inoculated with fresh suspensions of *P. xanthii* conidia (1 × 10^5^ conidia mL^−1^) after a complete drying period. As a negative control, discs treated with dsRNA obtained from an empty vector were used. Disease severity expressed as the percentage of leaf disc surface covered by powdery mildew, was recorded 8 d postinoculation (dpi). The data shown represent the mean value of 36 samples from three independent experiments, with error bars depicting the standard error. Data points followed by the same letter are not significantly different at *p* = 0.05 according to Fisher´s least significant difference test (LSD). (**B**) Molecular estimation of *P. xanthii* biomass on dsRNA-infiltrated melon cotyledons. Samples were taken at 72 h postinoculation (hpi) and employed for genomic DNA isolation. The ratios of *P. xanthii* to melon cotyledon genomic DNA (Px/Cm gDNA) were determined by qPCR. As a negative control, discs treated with dsRNA obtained from an empty vector were used. Bars represent the mean ± standard error of three technical replicates from three different DNA samples, each from five pooled cotyledons. Data points with the same letter have not significantly different at *p* = 0.05 according to Fisher´s least significant difference test (LSD). (**C**) Efficacy of dsRNA-induced gene silencing of the *P. xanthii* and *C. melo* genes. Total RNA was isolated from dsRNA-infiltrated melon cotyledons at 24 hpi. The relative expression of *P. xanthii* and melon genes was analyzed by RT-qPCR, and the change in gene expression levels was represented as fold change between silencing constructs and empty vector sample pairs after expression normalization to the *P. xanthii* elongation factor 1-alpha gene *PxEF1* (MK249653) and the *C. melo* actin-7 gene *CmACT7* (XM_008462689.2), which were used as endogenous controls. Bars represent the means ± standard errors of three technical replicates from three different samples, each from five pooled cotyledons. Data points followed by the same letter are not significantly different at *p* ≤ 0.05 according to Fisher´s least significant difference test (LSD).

To analyze the roles of these six *P. xanthii* conserved and non-annotated genes in plant-pathogen interactions, we first developed a new gene silencing method based on the application of exogenous dsRNA to the plant surface. For this purpose, we synthesized in vitro double-stranded RNA (dsRNA) molecules using the vector pL4440, which carries opposite T7 RNA polymerase promoters to amplify target sequences with the T7 promoter sequence at both ends, as previously described [15]. We initially produced dsRNAs for the melon gene *CmMlo1*, previously used as a positive control for RNAi-induced resistance against powdery mildews [30], and the *P. xanthii* genes *PxTUB2* and *PxCYP51*, whose gene products are the target proteins of methyl-benzimidazole carbamates (MBC fungicides; FRAC code 1) and demethylation inhibitors (DMI fungicides; FRAC code 3), respectively [52] (Figure S1A). To test the effects of the application of exogenous dsRNAs on *P. xanthii* growth, melon cotyledon discs were exposed to different concentrations of dsRNAs and inoculated with *P. xanthii* conidia after a complete drying period (Figure S2A). Disease symptoms were considerably reduced in leaf discs treated with dsRNAs (Figure 3A). Furthermore, it was observed that the lowest concentration of dsRNA tested (100 ng mL^−1^) was sufficient to induce the strongest effects in all cases.

To validate these results, we carried out similar experiments using melon cotyledons in which dsRNAs were applied by infiltration (Figure S2B). Twenty-four hours after dsRNA infiltration, cotyledons were inoculated with *P. xanthii* conidia. In this case, fungal biomass was molecularly estimated by qPCR 72 h after pathogen inoculation (Figure 3B). As expected, fungal growth was dramatically reduced by the infiltration of dsRNAs, corroborating the results obtained in leaf disc assays. In addition, it was observed that the lowest concentration of dsRNA (100 ng mL^−1^) was sufficient to induce the strongest fungal growth reductions. Furthermore, this reduction in fungal growth could be attributed to a decrease in the expression of the target genes by approximately 50% in all cases and concentrations (Figure 3C), a reduction similar to that previously described for *Agrobacterium tumefaciens*-mediated host-induced gene silencing (ATM-HIGS) assays [30,31,32]. Altogether, our results showed that foliar application of exogenous dsRNA could also be an efficient gene silencing method in *P. xanthii* and that application of dsRNA may have a strong impact on fungal growth and disease symptoms when the expression of an essential gene is considerably reduced.

### 3.3. CNAP948, CNAP1048, CNAP8878, CNAP9066, CNAP10905 and CNAP30520 Are Essential Proteins for P. xanthii

**Figure 4:**
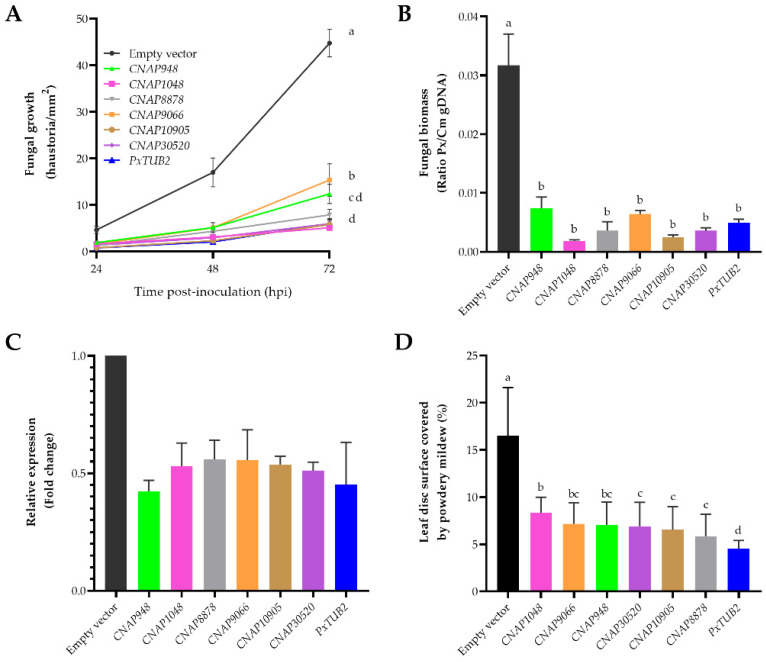
Effect of application of *CNAP*-based dsRNAs on *P. xanthii* growth and disease development. (**A**) Time course analysis of the formation of *P. xanthii* haustoria per unit area (mm^2^) on melon cotyledons after silencing of *P. xanthii CNAP* genes by dsRNA infiltration. Haustorial count was performed using an optical microscope and the leaf discs obtained with the DAB uptake method. Data represent the mean value of 30 samples from three independent experiments ± standard error. (**B**) Molecular estimation of *P. xanthii* biomass on dsRNA-infiltrated melon cotyledons. Samples were taken at 72 hpi and then used for genomic DNA isolation. The ratios of *P. xanthii* to melon cotyledon genomic DNA (Px/Cm gDNA) were evaluated by qPCR. (**C**) Efficacy of dsRNA-induced gene silencing of *P. xanthii CNAP* genes. Total RNA was isolated from dsRNA-infiltrated melon cotyledons at 24 hpi. The relative expression of *P. xanthii* genes was analyzed by RT-qPCR, and the change in gene expression levels was represented as fold change between silencing constructs and empty vector sample pairs after normalization to the expression of the endogenous control the *P. xanthii* elongation factor 1-alpha gene *PxEF1* (MK249653). As a positive control, *PxTUB2*-dsRNA was used. As a negative control, dsRNA obtained from an empty vector was used. In all cases, the dose of dsRNA used was 100 ng mL^−1^. Bars indicate the means ± standard errors of three technical replicates from three different samples, each from five pooled cotyledons. (**D**) Effect of silencing of *P. xanthii CNAP* genes on disease development. The results of a leaf disc assay conducted essentially as described in Figure 3A are shown. Data represent the mean values of 36 samples from three independent experiments, with error bars depicting the standard error. Data points with the same letter have not significantly different at *p* = 0.05 according to Fisher´s least significant difference test (LSD).

Once the efficacy of the application of dsRNA for the silencing of *P. xanthii* genes had been demonstrated, we proceeded to analyze the roles of the six selected CNAPs in fungal development and disease establishment by dsRNA-mediated gene silencing. For this purpose, we used cotyledon infiltration and leaf disc assays, in which a single concentration of dsRNA (100 ng mL^−1^) and dsRNA of *PxTUB2* were used as positive controls for fungal growth inhibition. First, cotyledon infiltration assays were conducted with dsRNAs produced for the different *CNAP* genes (Figure S1B). To quantify fungal growth after silencing of *P. xanthii* CNAP-coding genes, two approaches were used: haustorial counts by light microscopy and a molecular approach by qPCR. For haustorial counts, cotyledon discs were prepared for histochemical detection of hydrogen peroxide. The haustoria were identified as dark brown spots along the hyphae and inside epidermal cells. All *CNAP* genes were essential for fungal development. *CNAP1048*, *CNAP10905* and *CNAP30520* showed the highest contributions to fungal growth, and gene silencing highly reduced the number of haustoria to similar levels to *PxTUB2* gene silencing (Figure 4A). Similarly, the qPCR approach corroborated the results obtained by haustorial counts, showing that fungal growth was strongly affected by silencing of *P. xanthii CNAP* genes (Figure 4B). The efficacy of infiltration of dsRNAs in reducing *CNAP* gene expression was also studied. The results showed that the levels of *CNAPs* and *PxTUB2* transcripts decreased by approximately 50% in these assays (Figure 4C), a reduction similar to that previously observed. Finally, similar results were obtained in leaf disc assays (Figure 4D), indicating that these *CNAP* genes may have relevant roles in fungal development and suggesting that they could be promising targets for the management of powdery mildews by SIGS.

### 3.4. Efficient Control of Cucurbit Powdery Mildew by SIGS

**Figure 5:**
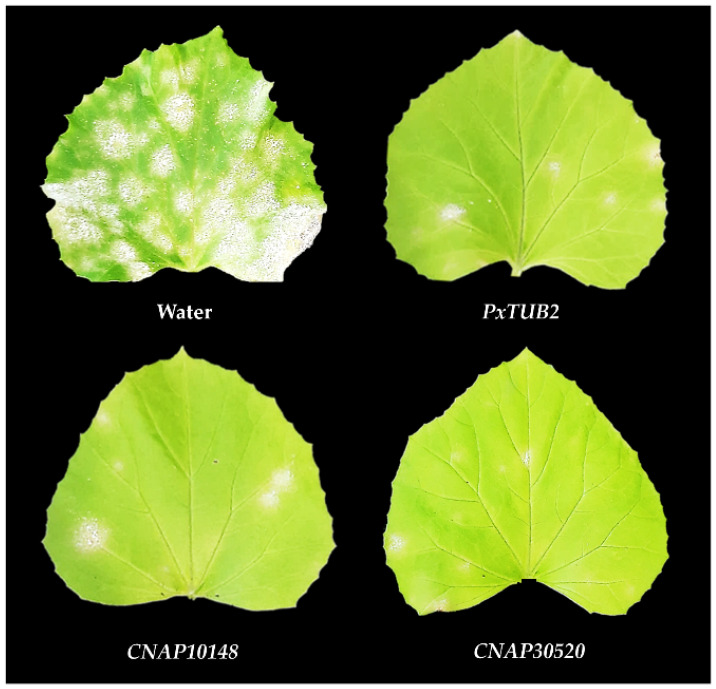
Reduction of powdery mildew symptoms in melon plants after spraying *CNAP*-based dsRNAs and induction of gene silencing (SIGS). Melon plants were sprayed with 20 µg mL^−1^ dsRNAs designed to suppress the expression of *P. xanthii CNAP* genes and inoculated with fresh suspensions of *P. xanthii* conidia (1 × 10^4^ conidia mL^−1^) 24 h after dsRNA application. Pictures were taken 12 days after inoculation of the fungal pathogen. Pictures: Water, leaf taken from a plant sprayed with water (negative control), showing the upper surface covered by powdery mildew. *PxTUB2*, leaf taken from a plant sprayed with *PxTUB2*-dsRNA (positive control), showing a dramatic reduction in powdery mildew symptoms. *CNAP1048* and *CNAP30520*, leaves taken from plants sprayed with *CNAP1048*-dsRNA and *CNAP30520*-dsRNA, respectively, showed disease symptom reductions similar to the positive control. Pictures are representative of the results shown in Table 3.

To explore the possibility of powdery mildew management by spray-induced gene silencing (SIGS), we selected three *CNAP* genes to conduct SIGS assays in plant growth chambers. For this purpose, second leaves of melon plants were sprayed with different concentrations of dsRNAs ranging from 5 to 30 µg mL^−1^ to target the *CNAP1048*, *CNAP10905* and *CNAP30520* genes, which have been shown to be critical for *P. xanthii* development in leaf discs and infiltration assays. Twenty-four hours after dsRNA spray applications, fresh suspensions of *P. xanthii* conidia were used to inoculate melon leaves. As shown in Table 3, dsRNA application produced significant negative effects on fungal growth with a drastic reduction in disease symptoms in terms of leaf surface covered by powdery mildew. In all cases, disease severity was reduced by approximately 80% to 90% compared with melon leaves treated with water. The best results were obtained with the highest concentrations of dsRNA tested, 20 and 30 µg mL^−1^ (Table 3; Figure 5). These results suggested that SIGS could be a novel and accurate approach for controlling cucurbit powdery mildew. 

## 4. Discussion

The loss of efficacy of most commonly used anti-powdery mildew fungicides [11] has created an urgent need to develop alternative approaches to fungicide applications to reduce the risks of environmental contamination issues and chances for pathogens to become resistant to fungicides. One of the most studied approaches is so-called host-induced gene silencing (HIGS), a promising RNAi-based technology in which genetically modified host plants express dsRNAs or sRNAs, which target pathogen virulence-related genes to fight against plant diseases [53,54]. Compared with chemical treatments, HIGS is a friendly alternative in plant protection because it combines high selectivity for the target organism with minimal side effects; however, it is restricted to plants with established transformation methods, limiting the number of crop plants in which this alternative can be employed [55,56]. This limitation can be overcome by SIGS, another RNAi-based technology that allows the inhibition of pathogens and disease development by topical application of sRNAs or dsRNA molecules onto plants to silence essential pathogen genes, conferring efficient and sustainable crop protection [13,57,58]. Thus, for example, spraying dsRNAs silencing *Fusarium graminearum* cytochrome P450 lanosterol C-14α-demethylase *(CYP51)* genes, which are required for the biosynthesis of fungal ergosterol, significantly reduced scab symptoms on barley leaves [13]. Similarly, spraying dsRNAs that target the Dicer-like protein 1 and 2 genes of *B. cinerea* on the surface of fruits, vegetables and flowers effectively suppressed grey mold diseases [14]. These and other studies suggest that such an RNAi-based disease control strategy is effective for both monocot and dicot crop species [17]. 

In this work, we examined the potential of RNAi-based spraying technology to control cucurbit powdery mildew as an alternative to conventional fungicides for effective disease management. As a first step, we evaluated the effect of exogenous dsRNA on *P. xanthii* growth using dsRNAs to silence plant and fungal genes, which affect fungal development. The negative effect on fungal growth was very evident in both leaf disc and cotyledon infiltration assays; dsRNA-treated melon cotyledon discs showed a considerable reduction in disease severity when treated with a low dose of dsRNA as well as dsRNA-infiltrated melon cotyledons, achieving a disease reduction of approximately 60 to 80%. Interestingly, once optimal gene silencing conditions were achieved, higher doses of dsRNA did not elicit a stronger silencing phenotype, as reflected in previous work [15]. Additionally, regarding gene silencing constructs, dsRNA targeting the β-tubulin gene, which encodes the target protein for MBC fungicides [52], proved to be one of the most effective dsRNAs tested, with *PxTUB2*-dsRNA selected as a positive control in dsRNA assays. As expected, empty vector dsRNA had no effect on fungal development or gene expression. Considering that a reduction in gene expression of approximately 50% obtained by the application of exogenous dsRNA was similar to that obtained by *Agrobacterium*-mediated HIGS [30,31,32], this strategy could be used as a new and simple gene silencing method in *P. xanthii*.

In the present study, we were able to identify a group of nonsecreted proteins without functional annotation or known domains, which were designated conserved and non-annotated proteins (CNAPs). The transcriptome of *P. xanthii* contains transcripts encoding sixty CNAPs, which were characterized by their cytoplasmic location. Although these CNAPs are highly conserved, especially in plant fungal pathogens, it was not possible to predict their functions based on sequence homology. However, in recent years, the development of in silico analyses combining different computational tools has substantially contributed to the prediction of the structures and functions of non-annotated proteins [59]. Protein modeling results suggested that six of these CNAPs could have a relevant role in fungal development, showing significant structural analogy with proteins involved in important biological processes. Interestingly, four of these CNAPs exhibited high structural analogy with respiration-related proteins. In particular, CNAP8878 and CNAP30520 presented significant structural analogy with subunits of yeast NADH-ubiquinone oxidoreductase as well as putative interactions with iron-sulfur clusters, suggesting that these CNAPs may be involved in respiration through the transfer of electrons from NADH to ubiquinone during oxidative phosphorylation. Furthermore, CNAP9066 and CNAP10905 also appear to be related to respiration because their structure and ligand predictions indicated potential activity as subunits of cytochrome c oxidase and ATP synthase, respectively. On the other hand, CNAP948 seems to be a drug efflux transporter. In the wheat powdery mildew fungus *Blumeria graminis* f. sp. *tritici*, a putative ABC transporter (BgABC1) is involved in the protection of the fungus against fungicide toxicity [60]. Based on this, fungal ABC transporters may be a strategy by which the pathogen becomes resistant to fungicides or antifungal compounds produced by plants, thus favoring disease establishment. Finally, regarding CNAP1048, its structure and ligand prediction data showed its potential activity as a glucosyltransferase, an enzymatic activity required for glycosylation in the N-linked glycosylation pathway. Protein N-glycosylation is one of the main known posttranslational modifications in eukaryotes, inducing correct folding and preventing proteolytic degradation [61].

Although the activity of these proteins has not been addressed in this work, the high quality of the protein models and the results of the other prediction tools, led us to hypothesize that these proteins should have important roles in determining the biology of *P. xanthii*, and we performed analysis by dsRNA-mediated gene silencing. The silencing of *P. xanthii CNAP* genes by dsRNA application resulted in significant reductions in fungal growth and disease symptoms, achieving reduced expression of target genes by approximately 50%, as previously reported [62]. Considering the impact on fungal development, these silencing phenotypes were classified as having a strong effect, according to the categories previously proposed [30]. These highly effective silencing phenotypes could be attributed to their putative functions; however, the precise contributions of these CNAPs to fungal development remain to be elucidated in future research. Convergent evolution is a known phenomenon in fungi, and the evolution of fungal pathogenicity genes is one of the most obvious examples [63,64]. Regarding basic cellular functions, one of the latest examples is the identification of convergent genes that shape budding yeast pericentromeres [65]. Although the biochemical activities of these CNAP proteins have not been demonstrated, it is tempting to speculate on the recruitment of convergent genes for the maintenance of essential physiological functions such as respiration or glycosylation.

Due to their important contributions to fungal development, the *CNAP1048*, *CNAP10905* and *CNAP30520* genes were selected as targets to evaluate the efficacy of SIGS for controlling cucurbit powdery mildew. In line with previous results, the spray application of these dsRNAs on melon leaves induced high levels of disease control. Moreover, we tested the efficacy of SIGS using various doses of dsRNA, and our data indicated that such dsRNAs remained functional at concentrations as low as 5 µg mL^−1^, although higher concentrations of dsRNA seem to provide higher disease control, as previously demonstrated [14]. Furthermore, the levels of disease reduction were very similar for all dsRNAs tested, indicating that these CNAPs are essential for fungal viability and could be interesting candidates for testing the efficacy of SIGS under greenhouse conditions.

To summarize, in this study, we have demonstrated that the spray application of dsRNA targeting *P. xanthii* genes effectively reduces the development of the pathogen and the progression of powdery mildew disease, supporting the idea that SIGS technology could also be used to control powdery mildews in a sustainable and environmentally friendly manner. Future studies should focus on improving the duration and effectiveness of spray application of dsRNA for cucurbit powdery mildew management under field conditions to characterize the commercial potential of SIGS. In addition, we have provided evidence that these conserved and non-annotated fungal proteins constitute an untapped group of potential target candidates against the cucurbit powdery mildew pathogen, and they could also be targets in other species of phytopathogenic fungi.

## Figures and Tables

**Table 1:** Features of *Podosphaera xanthii* conserved and non-annotated proteins (CNAPs) used in this study.

Sequence ID	Protein Name	Protein Length ^1^	Subject ID	Description	E-Value	Identity
Pxanthii_948	CNAP948	708	XP_007290516	Hypothetical protein *Marssonina brunnea* f. sp. multigermtubi MB_m1	0.0	80% (571/711)
Pxanthii_1048	CNAP1048	421	KAE8449429	Hypothetical protein *Helotiales* sp. DMI_Dod_QoI	0.0	81% (342/421)
Pxanthii_8878	CNAP8878	103	RKF57155	Hypothetical protein *Oidium neolycopersici*	7 × 10^−58^	81% (83/103)
Pxanthii_9066	CNAP9066	122	CCU82200	Hypothetical protein *Blumeria graminis* f. sp. *hordei* DH14	1 × 10^−59^	75% (92/122)
Pxanthii_10905	CNAP10905	74	ESZ98542	Hypothetical protein *Sclerotinia borealis* F-4128	8 × 10^−31^	77% (51/66)
Pxanthii_30520	CNAP30520	80	TVY46286	Hypothetical protein *Lachnellula occidentalis*	3 × 10^−47^	88% (70/80)

^1^ Number of amino acids in mature protein.

**Table 2:** Summary of data and predicted features of CNAPs obtained after structure modeling.

	Score Values ^1^		Structural Analogs ^2^		Predicted Features ^3^		
Protein Model	C Score	TM Score	PDB Code	Species	Activity	Ligands	Putative Function ^4^
CNAP948	−0.63	0.781	6TQE	*Mycobacterium tuberculosis*	Efflux transporter	ATP	Efflux transport
CNAP1048	−0.82	0.873	6SNH	*Saccharomyces cerevisiae*	Glucosyltransferase	Glucose	Glycosylation
CNAP8878	−1.22	0.689	6RFQ	*Yarrowia lipolytica*	Oxidoreductase	Iron-sulfur	Respiration
CNAP9066	−1.49	0.710	1XME	*Thermus thermophilus*	Oxidoreductase	Copper	Respiration
CNAP10905	0.37	0.624	5LQX	*Pichia angusta*	ATP synthase	ADP	Respiration
CNAP30520	0.80	0.839	6Y79	*Yarrowia lipolytica*	Oxidoreductase	Iron-sulfur	Respiration

^1^ Score values represent the quality of each model according to I-TASSER. C-score values, in the range of −5 to 2, measure the confidence of each model, representing a higher value a higher confidence. TM-score with values >0.5 indicate a correct topology. ^2^ Protein structurally close to three-dimensional models of *P. xanthii* proteins according to I-TASSER prediction. PDB, Protein Data Bank. ^3^ Summary results obtained from the software tools CATH/Gene3D, COACH, 3DLigandSite and Motif Scan. ^4^ Interpretation of the results obtained from the protein model and the predicted features.

**Table 3:** Efficacy of spray-induced gene silencing (SIGS) for the control of powdery mildew of cucurbits under plant growth chamber conditions.

Treatment	Dose of dsRNA (µg mL^−1^)	Leaf Surface Covered by Powdery Mildew (%) ^1^	Disease Reduction (%) ^2^
Water	-	59.35 ± 3.78 a	-
Empty vector-dsRNA	30	55.60 ± 4.75 a	6.32
*CNAP1048*-dsRNA	30	5.62 ± 1.01 ef	90.53
	20	5.25 ± 0.72 ef	91.15
	10	10.04 ± 0.58 cde	83.08
	5	21.26 ± 1.28 b	64.18
*CNAP30520*-dsRNA	30	3.92 ± 0.62 f	93.40
	20	3.74 ± 0.46 f	93.70
	10	9.11 ± 0.78 cdef	84.65
	5	14.83 ± 1.26 c	75.01
*CNAP10905*-dsRNA	30	3.41 ± 0.28 f	94.25
	20	3.53 ± 0.26 f	94.05
	10	8.24 ± 1.51 def	86.12
	5	12.96 ± 1.49 cd	78.16
*PxTUB2*-dsRNA	30	3.86 ± 0.23 f	93.50
	20	4.51 ± 0.45 ef	92.40
	10	11.94 ± 1.89 cd	79.88
	5	22.83 ± 0.60 b	61.53

^1^ Disease severity was represented as the percentage of leaf surface covered by powdery mildew at 12 d postinoculation (dpi). As a negative control, melon leaves treated with water were used. Data represent the mean value of 18 samples from three independent experiments ± standard error. Values with the same letter have not significantly different at *p* = 0.05 according to Fisher´s least significant difference test (LSD). ^2^ Percentage of disease reduction achieved by each treatment referred to values of melon leaves treated with water.

## Data Availability

The sequence data are available in the supplementary material section (Table S2).

## Supplementary Materials

The following are available online at https://www.mdpi.com/article/10.3390/jof7090735/s1, Table S1: Primers used in this study, Table S2: List of CNAPs identified in the *P. xanthii* transcriptome. Figure S1: Production of dsRNAs. Figure S2: Representative images of leaf disc and cotyledon infiltration assays.
