# Supplementary material for: Gene Mining for Conserved, Non-Annotated Proteins of Podosphaera xanthii Identifies Novel Target Candidates for Controlling Powdery Mildews by Spray-Induced Gene Silencing
Source: J Fungi (Basel). 2021 Sep 8;7(9):735. doi: 10.3390/jof7090735 (PMC8465782; doi:10.3390/jof7090735)
Supplement: Supplementary file 1 [file jof-07-00735-s001.zip › jof-1361287-supplementary.pdf]

SUPPORTING INFORMATION

Table S1. Primers used in this study.

Table S2. List of CNAPs identified in the *P. xanthii* transcriptome.

Figure S1. Production of dsRNAs.

Figure S2. Representative images of leaf disc (A) and cotyledon infiltration (B) assays.

Table S1. Primers used in this study.

Primer name	Sequence (5′- 3′)[1]
**Plasmid construction**	
CNAP948F-si	5′-TAATCCATGGCCTTGGCGAATAGAGTTGGC-3′ (NcoI)
CNAP948R-si	5′- TAATAGATCTAGTCCTAGTGCTCCCCAAAC-3′(BglII)
CNAP1048F-si	5′-TAATCCATGGAAGGCGGTTCTGTTTACTG-3′ (NcoI)
CNAP1048R-si	5′-TAATAGATCTTCCTCGGTGAGGTGTAAAG-3′ (BglII)
CNAP8878F-si	5′-TAATCCATGGACTTGGGCAAGAAGAAGTG-3′ (NcoI)
CNAP8878R-si	5′-TAATAGATCTGCAGTCCCAGGTTCCTTATC-3′ (BglII)
CNAP9066F-si	5′-TAATCCATGGATTGGATCCCTCTCTGGTGC-3′ (NcoI)
CNAP9066R-si	5′-TAATAGATCTCCAGTGGTGTTACAGGTCCT-3′ (BglII)
CNAP10905F-si	5′-TAATCCATGGTGGAAAGCCTCAGGTCTTAC-3′ (NcoI)
CNAP10905R-si	5′-TAATAGATCTCGGACGCAGCAGAATTTAC-3′ (BglII)
CNAP30520F-si	5′-TAATCCATGGGTGTTCCATTCGAGGCTC-3′ (NcoI)
CNAP30520R-si	5′-TAATAGATCTGTCCTCTCAACCATCCAGT-3′ (BglII)
Mlo1F-si	5′-TAATCCATGGGAACGACGCTGGAATTCAC-3′ (NcoI)
Mlo1R-si	5′-TAATAGATCTGGCCGTACCAGAAATACTC-3′ (BglII)
TUB2F-si	5′-TAATCCATGGATGTTGTTCGGCGTGAAGC-3′ (NcoI)
TUB2R-si	5′-TAATAGATCTGAGGTCGCCATATGAAGGG-3′ (BglII)
CYP51F-si	5′-TAATCCATGGAAGCTATGGCCCAGATCAC-3′ (NcoI)
CYP51R-si	5′-TAATAGATCTCGGGCACTGGAGTTCCATC-3′ (BglII)
**Gene expression analyses**	
CNAP948-F	5′-CTTTGGCCCGTTTATGGAGG-3′
CNAP948-R	5′-CGGTAACCCCTTTTCTTCGC-3′
CNAP1048-F	5′-GTCTTCCTATCCAGCAGGATTG-3′
CNAP1048-R	5′-TGCCTATAGAGGGACAGCATTC-3′
CNAP8878-F	5′-TGTCTCCCGTTGTTTTCCATT-3′
CNAP8878-R	5′-CGATTCTATGAGCCTCTTTCTTG-3′
CNAP9066-F	5′-TCAGATGCTACGAGGAGAACA-3′
CNAP9066-R	5′-ACTGCCGCTAAACATGATCA-3′
CNAP10905-F	5′-TTTCGCGTGGAAAGCCTCAG-3′
CNAP10905-R	5′-GCTTGCCATGACTCCATTTAGC-3′
CNAP30520-F	5′-TGTTCCATTCGAGGCTCTCA-3′
CNAP30520-R	5′-CGATCCATCATTTGCCTATCCC-3′
Mlo1-F	5′-TCACAGGCTGGTTATTGTGC-3′
Mlo1-R	5′-GCGAATCTTCAATCCTCCAA-3′
TUB2-F	5′-TGACCGTCCCTGAATTAACC-3′
TUB2-R	5′-GAACGTTGTTGGGAATCCAT-3′
CYP51-F	5′-GGTGCATCAAGCCAGTACCT-3′
CYP51-R	5′-CAGTCTCTCTGACCCCTTGC-3′
**Molecular estimation of fungal biomass**	
TUB2g-F	5′-TTGTAGGAATCACATCCCTTTCTC-3′
TUB2g-R	5′-TTCTTCCGGTTGCATGGGTGGTTC-3′
Acting-F	5′-GGCTGGATTTGCCGGTGATGATGC-3′
Acting-R	5′-GGAAGGAGGAAATCAGTGTGAACC-3′

[1]Underlined sequence denotes the recognition site for the restriction enzyme (marked in brackets).

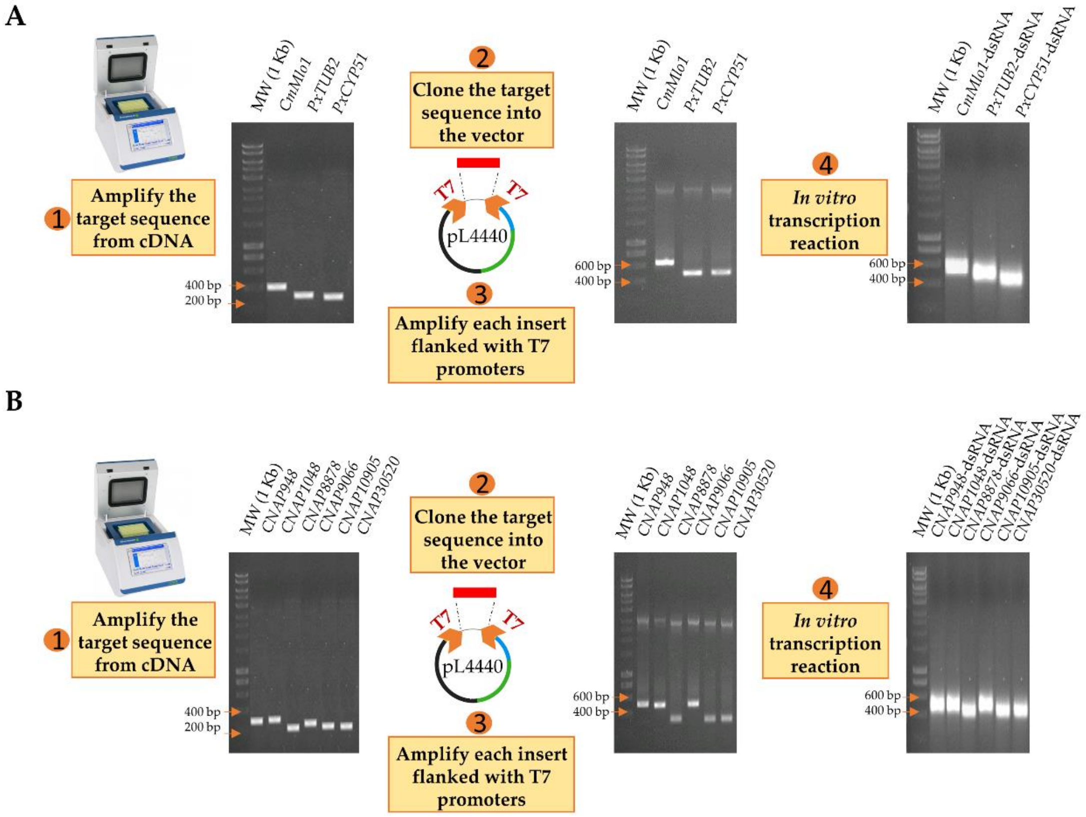

Figure S1. Production of dsRNAs. **(A)** Schematic representation of *in vitro* production of dsRNAs targeting *CmMlo1*, *PxTUB2* and *PxCYP51* genes. **(B)** Schematic representation of *in vitro* production of dsRNAs targeting *CNAP* genes.

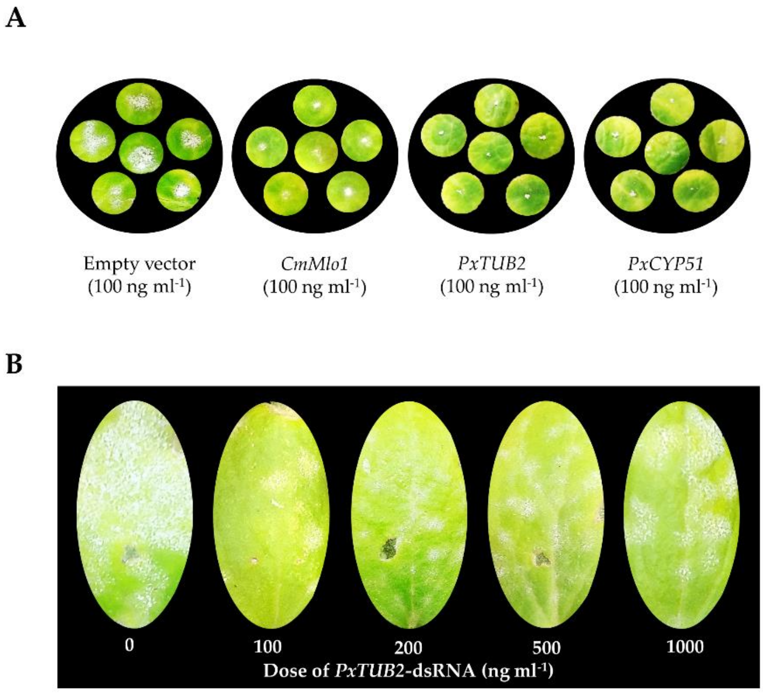

Figure S2. Representative images of leaf disc and cotyledon infiltration assays. **(A)** Melon cotyledon discs were exposed to 100 ng ml^{-1} dsRNAs designed to suppress the expression of *CmMlo1*, *PxTUB2* and *PxCYP51* genes and inoculated with fresh suspensions of *P. xanthii* conidia ($1{\cdot}10^5$ conidia ml^{-1}) after a complete drying period. As a negative control, discs treated with dsRNA obtained from an empty vector were used. Pictures were taken 8 days after inoculation of the fungal pathogen. Fungal growth and disease symptoms were considerably reduced in leaf discs treated with dsRNAs. Pictures are representative of the results shown in Figure 3A. **(B)** Melon cotyledons were infiltrated with different doses of *PxTUB2*-dsRNA and inoculated with fresh suspensions of *P. xanthii* conidia ($1{\cdot}10^5$ conidia ml^{-1}) 24 h after dsRNA application. Pictures were taken 12 days after inoculation of the fungal pathogen. Fungal growth was dramatically affected by the infiltration of dsRNAs. Pictures are representative of the results shown in Figure 3B.
